# Treatment of 13-cis retinoic acid and 1,25-dihydroxyvitamin D3 inhibits TNF-alpha-mediated expression of MMP-9 protein and cell invasion through the suppression of JNK pathway and microRNA 221 in human pancreatic adenocarcinoma cancer cells

**DOI:** 10.1371/journal.pone.0247550

**Published:** 2021-03-17

**Authors:** Yen-Huang Cheng, En-Pei Isabel Chiang, Jia-Ning Syu, Che-Yi Chao, Hung-Yu Lin, Cheng-Chieh Lin, Mei-Due Yang, Shu-Yao Tsai, Feng-Yao Tang

**Affiliations:** 1 Department of Emergency Medicine, Show Chwan Memorial Hospital, Changhua, Taiwan; 2 Department of Food Science and Biotechnology, National Chung Hsing University, Taichung, Taiwan; 3 Innovation and Development Center of Sustainable Agriculture (IDCSA), National Chung Hsing University, Taichung, Taiwan; 4 Biomedical Science Laboratory, Department of Nutrition, China Medical University, Taichung, Taiwan; 5 Department of Food Nutrition and Health Biotechnology, Asia University, Taichung, Taiwan; 6 Department of Medical Research, China Medical University Hospital, China Medical University, Taichung, Taiwan; 7 Research Assistant Center, Show Chwan Memorial Hospital, Changhua, Taiwan; 8 School of Medicine, College of Medicine, China Medical University, Taichung, Taiwan; 9 Department of Family Medicine, China Medical University Hospital, Taichung, Taiwan; 10 Department of Healthcare Administration, College of Health Science, Asia University, Taichung, Taiwan; 11 Department of Surgery, China Medical University Hospital, Taichung, Taiwan; Duke University School of Medicine, UNITED STATES

## Abstract

Human pancreatic ductal adenocarcinoma (PDAC) is a deadly cancer type with a very high mortality rate. Inflammatory cytokine such as tumor necrosis factor- alpha (TNF-α) plays a pivotal role in the progression of PDAC. Recently, suppression of cell invasion by preventive agents has received considerable attention in the prevention of metastatic tumors. Several clinical studies suggested that natural forms or analogues of fat-soluble vitamins such as vitamin A and vitamin D can work as anti-cancer agents to inhibit the development of cancer. In this study, our results demonstrated that co-treatment of 13-cis retinoic acid (13-cis RA) and 1,25-dihydroxyvitamin D3 (1,25-VD3) significantly inhibited TNF-α mediated cell invasion in PDAC *in vitro*. Cotreatment of 13-cis RA and 1,25-VD3 also inhibited TNF-α mediated expression of matrix metalloproteinase-9 (MMP-9) protein through blocking c-Jun N-terminal kinase (JNK) and nuclear factor kappa B (NF-κB) signaling pathways. Our results demonstrated that treatment of TNF-α lead to a decreased expression of tissue inhibitor of metalloproteinase- 3 (TIMP-3) protein and an induction of MMP-9 protein and cell invasion through an upregulation of microRNA-221 (miR-221) in human PDAC cells. Moreover, treatment of SP600125 (a specific inhibitor of JNK pathway) or cotreatment of 13-cis RA and 1,25-VD3 significantly induced a decreased expression of miR-221 and an increased expression of TIMP-3 protein. These results suggest that 13-cis RA and 1,25-VD3 significantly suppress TNF-α mediated cell invasion and therefore potentially act as preventive agents against PDAC.

## Introduction

Pancreatic cancer is a deadly cancer type and featured with extremely poor prognosis in the world [1]. In most clinical condition, advanced-stage pancreatic cancer is commonly diagnosed with hepatic metastasis [1]. Human pancreatic cancer is classified as two major types: exocrine and neuroendocrine cancers. Pancreatic ductal adenocarcinoma (PDAC), one of exocrine pancreatic cancer subtypes, mainly accounts about 85% of pancreatic cancer cases and tends to be a very aggressive one [2]. Due to the lack of treatment options and early stage- diagnostic biomarkers, PDAC patients usually have a very low 5-year survival rate around 8% [1, 2].

Several studies indicate that cancer microenvironment such as cytokines and matrix metalloproteinases (MMPs) play important roles in the progression and metastasis of pancreatic cancer [3]. Among these cytokines, tumor necrosis factor- alpha (TNF-α) is a key inflammatory cytokine and involves in the inflammatory response during tumor development [4]. A previous study suggests that TNF-α is also important in determining cell invasion of cancer cells [5]. TNF-α induces an activation of corresponding TNF-α receptor (TNF-α R), various downstream signaling pathways including the c-Jun N-terminal kinase (JNK) and nuclear factor- kappa B (NF-κB) pathways and subsequent epithelial-mesenchymal transition (EMT) in many types of cancer cells [6, 7]. Clinical studies have shown that pancreatic cancer patients have higher serum levels of TNF-α in comparison with normal subjects [8]. These findings suggest that a high expression of inflammatory cytokines such as TNF-α is correlated to pathological stages of pancreatic cancer [9].

Several studies indicate that an overexpression of MMPs such as MMP-9 is highly correlated with tumor progression including invasion, angiogenesis and metastasis in pancreatic cancer [10, 11]. A study indicates that a loss of TIMP3 is associated with tumor invasion in experimental animals [12]. It is already known that the tissue inhibitor of metalloproteinase 3 (TIMP3) could act as an inhibitor of MMP-9 to block its proteolytic activity [13]. Several studies also reported that miRNA-221 (miR-221) is highly expressed in pancreatic cancer patients and inversely correlated to survival rate [14–16]. A recent study indicated that miR-221 is upregulated by AP-1 transcription factor and involved in the downregulation of *TIMP3* gene during tumor development [17]. Another recent study also suggests that miR-221 promoted cell invasion through an up-regulation of MMP-9 [18]. These findings suggested that miR-221, TIMP3 and MMP-9 could represent as therapeutic targets of TNF-α-mediated cell invasion in PDAC cells.

Retinoids (active forms of fat-soluble vitamin A) and 1,25-dihydroxyvitamin D3 (1α,25(OH)_2_D_3_;1, 25-VD3; the active form of fat-soluble vitamin D) play important roles in the maintenance of cellular functions and human health [19, 20]. The major forms of retinoids refer to retinol and its natural metabolites or analogues include all-trans retinoic acid (ATRA), 9-cis retinoic acid (9-cis RA), 13-cis retinoic acid (13-cis RA). These retinoids involve in several important functions including gene regulation, cellular development, differentiation, proliferation and apoptosis in human epithelial cells [21]. A recent study showed that retinoid concentration is lower in PDAC tissue in comparison with the one in healthy subject [22]. Other studies also suggested that plasma level of vitamin D is negatively correlated to the incidence of pancreatic cancer [23, 24]. A study also indicated that low level of vitamin D receptor (VDR) was correlated with poor prognosis and survival rate in pancreatic cancer patients [25]. These evidences suggested that retinoids and vitamin D might play important roles in the prevention of tumor progression in advanced pancreatic cancer patients.

A recent study demonstrated that all-trans retinoic acid (ATRA) inhibited cellular matrix remodeling and inhibited cancer cell invasion [26]. Treatment of all-trans retinoic acid (ATRA) and gemcitabine exert synergistic effects on the blockade of cell survival in pancreatic cancer cells [27]. Several studies demonstrated anti-proliferation effects of ATRA, 9-cis-retinoic acid and vitamin D analogues in pancreatic cancer cells [28, 29]. To date, no findings have confirmed the preventive effects of 13-cis RA and 1, 25-VD3 on cell invasion and the expression of miR-221, MMP-9, TIMP-3 in PDAC cells. Due to the limited preventive and therapeutic tools to cancer metastasis, development of early prevention of metastasis is highly demanded in preclinical and clinical studies. Therefore, we investigated the chemo-preventive effects and mechanisms of action of 13-cis RA and 1, 25-VD3 on the prevention of cell invasion and MMP expression in PDAC cells.

## Materials and methods

### Antibodies, chemicals and reagents

We purchased the following antibodies including RelA/ p65 (NF-κB) (#3033T; Lot# 17), phospho-IκBα (Ser32/36) (#9246S; Lot# 16), p-JNK (Thr183/Tyr185) (#9251S; Lot# 11), E-cadherin (#5296S; Lot# 2), N-cadherin (#4061S; Lot# 3), Slug (#9585S; Lot# 2), and MMP-9 (#2270S; Lot# 2) from Cell signaling Technology (Danvers, MA, USA). Antibodies against phospho-c-jun (Ser63) (sc-822; Lot # L0717), Twist1 (sc-15393; Lot # F1109), TIMP3 (sc-373839; Lot # D2316), actin (sc-1616; Lot # L3004) and lamin A (sc-7292; Lot # L1919) were obtained from Santa Cruz Biotech Inc. (Dallas, TX, USA). The c-fos antibody (GTX129846; Lot # 42256) was purchased from GeneTex Inc (Irvine, CA, USA). Sodium bicarbonate, and dimethyl sulfoxide (DMSO) were purchased from Sigma-Aldrich (St Louis, MO, USA). We also purchased fetal bovine serum (FBS), Dulbecco’s Modified Eagle’s Medium (DMEM), 3-(4,5-dimethylthiazol-2-yl)-2,5-diphenyltetrazolium bromide) (MTT), NE-PER nuclear and cytoplasmic extraction reagent Kit and sodium-dodecyl sulfate (SDS) from Thermo Fisher Scientific (Waltham, MA, USA). Human tumor necrosis factor-α (TNF-α) recombinant protein was purchased from R&D Systems Inc. (Minneapolis, MN, USA).

### Cell culture

Authenticated human PDAC PANC-1 cell line (ATCC^®^ CRL-1469™) and HPAF-II (ATCC^®^ CRL-1997™) were acquired from American Type Culture Collection (Manassas, VA, USA) and provided by the laboratory of Dr. Wen-Hwa Lee of Genomics Research Center, Academia Sinica (Taiwan, Republic of China). Human PDAC PANC-1 and HPAF-II cells were cultured in 10% FBS DMEM. In this study, human PDAC cells were treated with TNF-α (50 ng/mL) in the presence or absence of 13-cis RA and 1, 25-VD3.

### Cell survival analysis

In this study, we measured cell viability by performing MTT assay. Human PDAC cells (2x 10^4^ cells/well) were cultured in 24- well plates and treated with TNF-α in the presence or absence of 13-cis-RA and 1, 25-VD3 for 24 hr. At the end of experiment, media were removed from each well of 24-well plates and replaced with MTT solution (0.5 mg/mL). After 1 hr incubation, MTT solution was discarded from each well and replaced with isopropanol to dissolve the purple depositor. About two hundred microliter of reaction solution was transferred into each well of 96-well plate for the measurement of optical density at 570 nm wave length with a multichannel plate reader.

### Analysis of cell invasion

To measure the cell invasion, human PDAC cells (5,000 cells/well) were cultured in the upper chamber coated with a Matrigel-coated filter in each transwell Boyden chambers. Human PDAC cells were stained with dye, Calcein AM, for monitoring cell invasion. These Calcein AM stained cells were then treated with 13-cis-RA or 1, 25-VD3 under the stimulation of TNF-α (50ng/ml) for 24 hr in DMEM medium (5000 cells /well). At the end of the experiment, non-invading cells and Matrigel in the upper chamber were removed by cotton swabs. Those invading cells at the lower side of the filter were fixed with 1% formaldehyde, stained with crystal violet staining solution. Invasive cells of each well were counted in 6 randomly selected fields under the Inverted Microscope (Olympus IX-71) (Olympus, Tokyo, Japan).

### Gelatin zymography

Gelatin zymography was performed to measure MMP-9 activity. Equal amount of proteins from supernatant was loaded into each well of 0.1% gelatin gel. After electrophoresis, the gel was washed with washing buffer for 1hr and zymograms were incubated with reaction buffer for another 24 hr. At the end of incubation, the gel removed from reaction buffer was stained with Comassie blue and rinsed with de-staining solution (7% methanol and 5% acetic acid). Band densities of clear zone represented as enzymatic activity and were quantitated by ImageJ software.

### Transfection of anti-miR-221 microRNA construct

Human PDAC cells were cultured in six-well plates and transfected with miRZip-221 anti-miR-221 microRNA construct (MZIP221-PA-1; System Biosciences, LLC) or control vector for 24 hr by using Lipofectamine 3000 Reagent (Thermo Fisher Scientific, Waltham, USA). At the end of experiments cells were collected for the invasion assay, measurement of protein expression for further analysis.

### Quantitative real-time PCR (qPCR)

Total RNA samples from human PDAC cells were collected by using RNAzol® RT reagent (RN 190; Molecular Research Center, Cincinnati, USA). RNA samples were converted into cDNA using TaqMan™ MicroRNA Reverse Transcription Kit (4366596; Applied Biosystems™) for the analysis of miRNA. These cDNA samples were reacted with hsa-miR-221 specific primers in a PCR reaction mix by using TaqMan™ Universal Master Mix II and TaqMan® MicroRNA Assays. An U6snRNA was used as the internal control. For the measurement of miR-221 expression, quantitative PCR experiments was performed using the CFX Connect™ Real-Time PCR Detection System (Bio-Rad Laboratories, Inc. California, USA). The expression level of miR-221 was adjusted by the U6 snRNA in PDAC cells and represented as a percentage of the untreated control subgroup.

### Preparation of protein extraction and Western blotting analysis

Cellular proteins (nuclear and cytoplasmic factions) were collected using NE-PER nuclear and cytoplasmic extraction reagent Kit according to the manufacturer’s instruction protocol. Cytoplasmic proteins and nucleus proteins were separately fractioned using 10% SDS- polyacrylamide gel electrophoresis (SDS-PAGE), transferred onto a PVDF membrane and blotted with primary antibodies such as anti-E-cadherin monoclonal antibody (mAb) and anti- NF-κB p65 mAb, respectively. The expression levels of phospho-IκBα (Ser32/36), phospho-JNK (Thr183/Tyr185), N-cadherin, TIMP3 and MMP-9 in cytoplasmic lysates were measured using the same procedure described above. The blots were stripped with washing buffer and reporbed with anti-actin mAb as the internal loading control. The expression levels of nuclear Slug, Twist1, c-fos and phospho-c-jun (Ser63) proteins were measured using the same procedure described above. The blots were stripped with washing buffer and reporbed with anti- lamin A mAb as the internal loading control.

### Biostatistical analysis

The quantitative methodology was used to determine whether difference between control and experimental subgroups exists in each individual experiment. The biostatistical analyses of the differences in this study were performed using SYSTAT software. A significant difference in cell invasion, zymogram or miR-221 expression among the subgroups was performed by analysis of variance followed by two-way ANOVA with Duncan post hoc test. Confirmation of a significant difference requires rejection of the null hypothesis of no difference between the mean obtained from sets of subgroups at the *P* = 0.05 level.

## Results

### 13-cis RA and 1,25-VD3 could mildly modulate the expression of EMT-regulatory proteins in human PDAC cells

**Fig 1:**
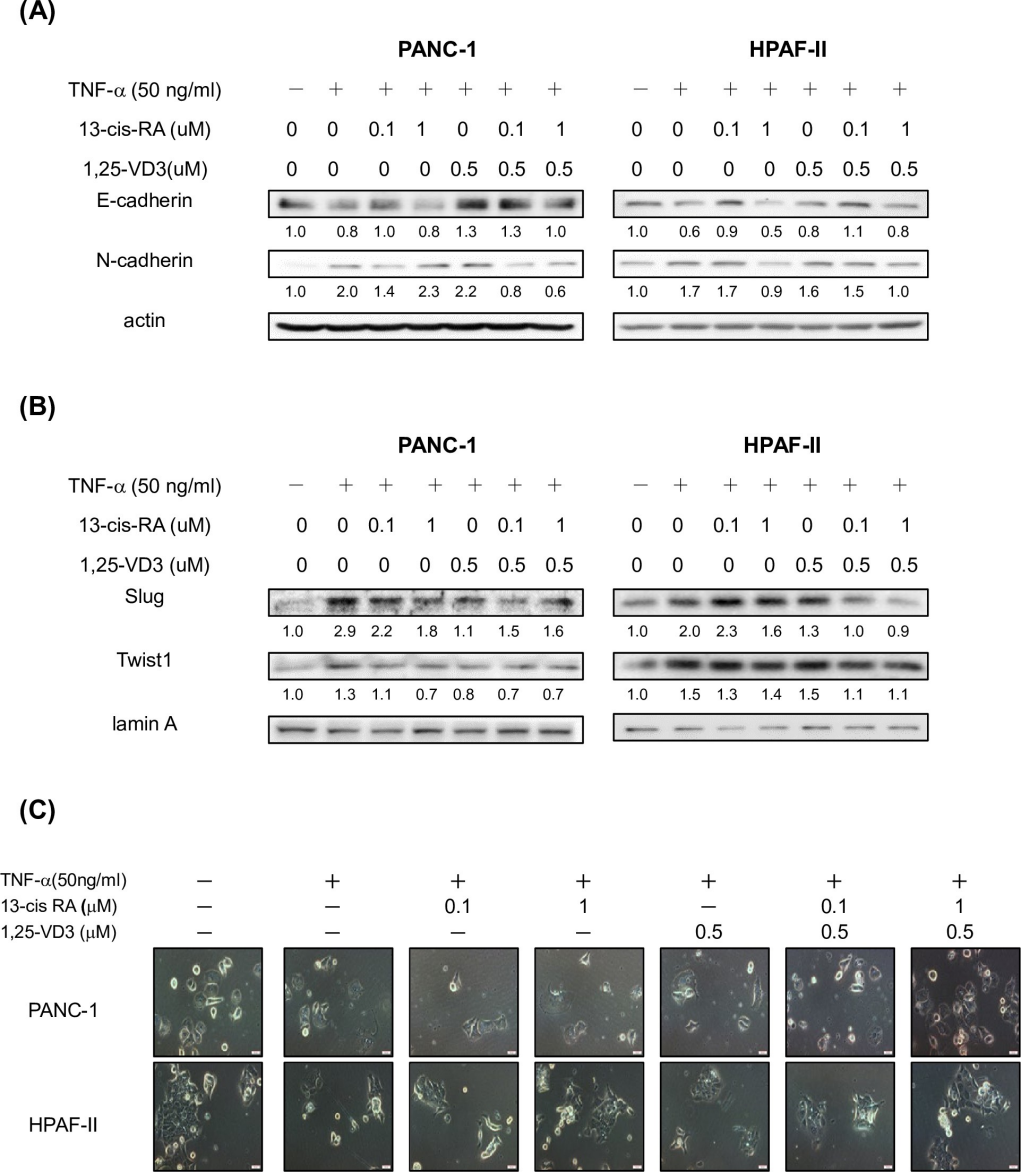
13-cis RA and 1,25-VD3 could mildly modulate the expression of EMT- regulatory proteins in human PDAC cells. Human PDAC PANC1 and HPAF-II treated with or without TNFα in 10% FBS DMEM for 24hr. Western blot analysis of cytoplasmic proteins (at 24 hr time point) using monoclonal antibodies against E-cadherin, N-cadherin, Slug, Twist1, actin and lamin A. Band intensities represent the amounts of E-cadherin and N-cadherin proteins in the cytoplasm (A) as well as the amounts of Slug and Twist1 proteins in the nuclei (B) of PDAC cells. (C)The morphology of human PDAC cells were observed under the inverted phase-contrast microscopy (300 x) and photomicrographs were documented with the Olympus DP-71 digital camera and imaging system (Tokyo, Japan).

Previous studies indicated that TNF-α is involved in the EMT and tumor progression in cancer cells [7]. Thus, we further investigated whether 13-cis RA and 1, 25-VD3 could suppress TNF-α-mediated expression of EMT-regulatory proteins in human PDAC cells. As shown in Fig 1A, Treatment of TNF-α lead to a reduced expression of E-cadherin protein and increment of N-cadherin protein in PDAC cells. Interestingly, treatment of 13-cis RA and 1, 25-VD3 mildly induced an increment of E-cadherin protein expression and a decreased expression of N-cadherin protein in TNF-α treated PDAC cells (Fig 1A). Our results also demonstrated that TNF-α enhanced the nuclear expression of EMT-regulatory proteins such as Slug and Twist1 in PDAC cells (Fig 1B). Our consistent findings also showed that treatment of 13-cis RA and 1, 25-VD3 suppressed the nuclear levels of Slug and Twist1 proteins in PDAC cells (Fig 1B). In Fig 1C, treatment of 13-cis RA and 1, 25-VD3 mildly changed the morphology of human PDAC cells upon TNF-α stimulation. These results suggested that treatment of 13-cis RA and 1, 25-VD3 could mildly reduce the expression of EMT- regulatory proteins such as Slug, Twist1 and N-cadherin and enhance the expression of E-cadherin protein in PDAC cells.

### 13-cis RA and 1,25-VD3 significantly inhibited TNF-α-mediated cell invasion in human PDAC cells *in vitro*

**Fig 2:**
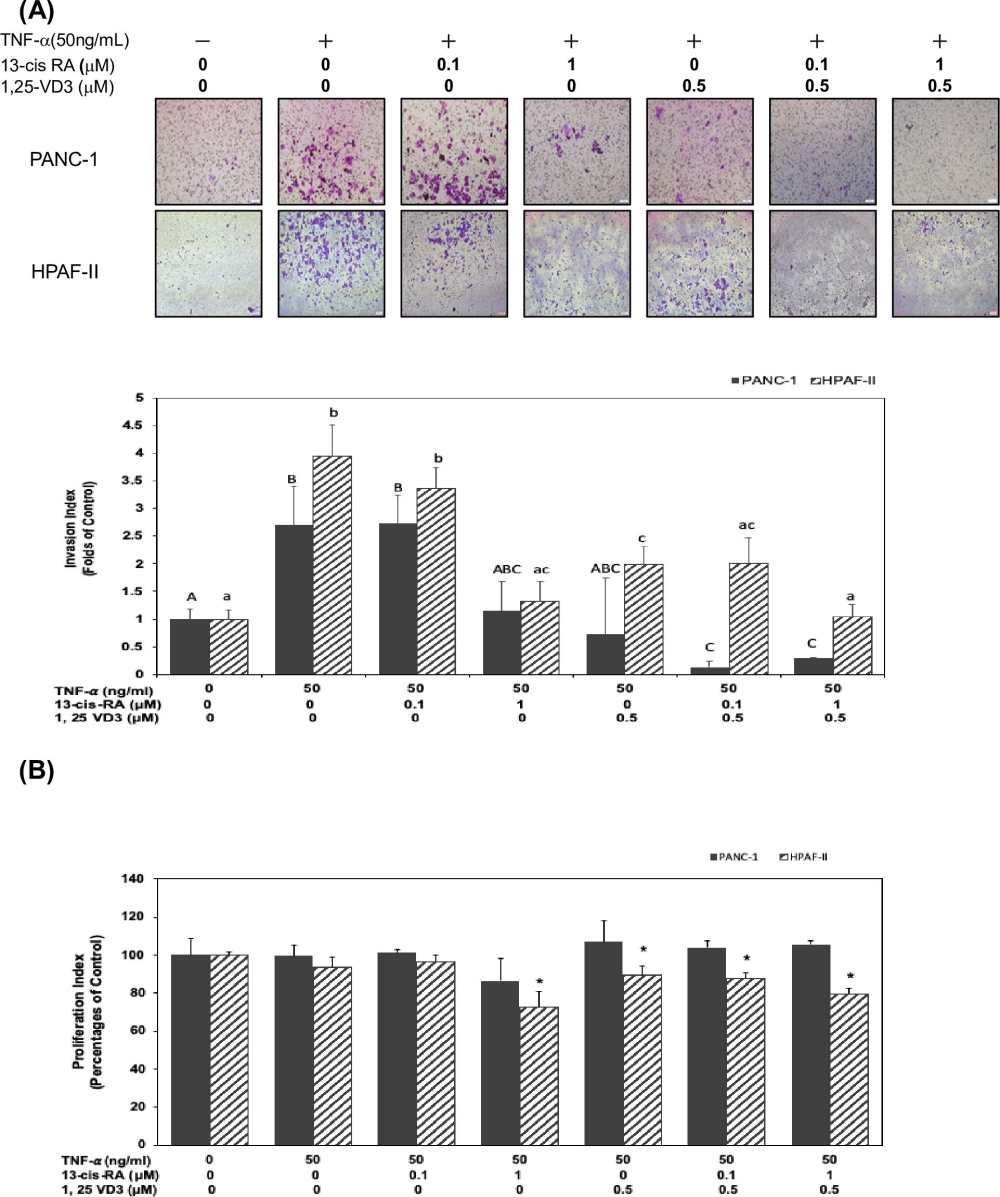
13- cis RA and 1,25-VD3 significantly inhibited TNF-α-mediated cell invasion in human PDAC cells *in vitro*. (A) Human PDAC PANC1 and HPAF-II cells were cultured in DMEM and treated with TNF-α in the presence of 13- cis RA or 1,25-VD3 for 24 hr. At the end of experiment, invasion of human PDAC cells was analyzed in Boyden chambers coating with Matrigel™ as described in Materials and Methods. Invasive human PDAC cells were counted as described above and the number of human PDAC cells invading into the lower side of the filter was measured as the invasion index. Different upper-case letters (A, B, C) represent statistically significant differences among subgroups in PANC-1 cells (*P*<0.05). Different lower-case letters (a, b, c) represent statistically significant differences among subgroups in HPAF-II cells (*P*<0.05). (B) Same conditions were performed to measure cell survival (at 24 hr time point) using MTT assay as described in Materials and Methods. Statistical significance is expressed as the mean ± SD (standard deviation) of three independent experiments. An asterisk (*) represent statistically significant differences in comparison with the untreated control subgroup(*P*<0.05).

We further investigated whether 13-cis RA and 1, 25-VD3 could modulate TNF-α-mediated cell invasion in human PDAC cells. Our results showed that TNF-α significantly induced cell invasion (Fig 2A) in human PDAC cells (*P*<0.05). Therefore, we further investigated whether treatment of 13-cis RA and 1, 25-VD3 could inhibit TNF-α-mediated cell invasion in human PDAC cells. As shown in Fig 2A, co-treatment of 13-cis RA (at concentration of 0.1 and 1 μM) and 1, 25-VD3 (at concentration of 0.5 μM) significantly inhibited cell invasion in human PDAC cells (*P*<0.05) (Fig 2A).

To examine whether the inhibitory effects of 13-cis-RA and 1, 25-VD3 on cell invasion were due to the cytotoxic effects, we measured cell viability by using MTT assay. As shown in Fig 2B, treatment of 13-cis RA and 1, 25-VD3 slightly affect cell survival rate in human PDAC HPAF-II cells but not in PANC-1 cells. These results suggested that 13-cis RA and 1, 25-VD3 might act as chemo-preventive agents to prevent cell invasion through targeting specific regulatory cascades in human PDAC cells.

### 13-cis RA and 1,25-VD3 inhibited TNF-α-mediated expression of MMP-9 protein through blockade of NF-κB and JNK signaling pathways

**Fig 3:**
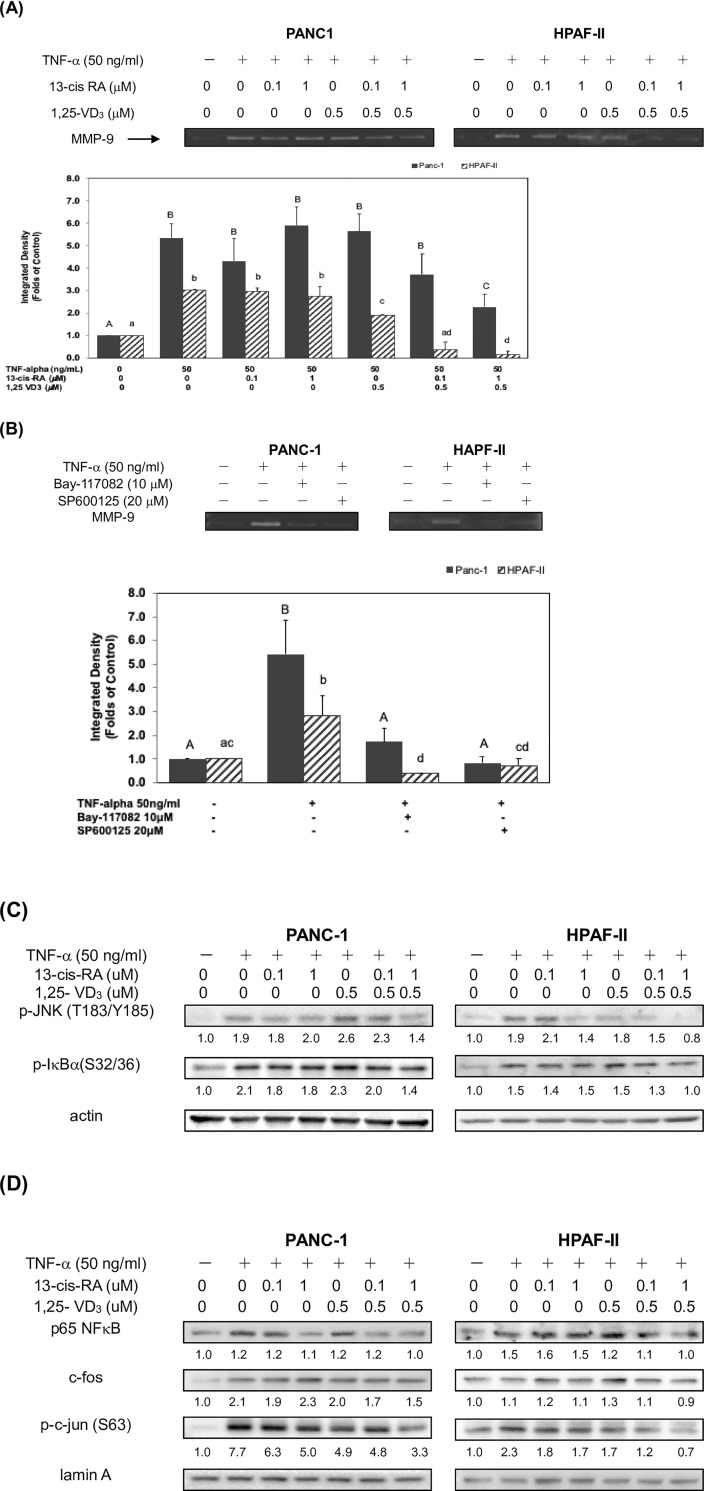
13-cis RA and 1,25-VD3 inhibited TNF-α-mediated expression of MMP-9 protein through blockade of NF-κB and JNK signaling pathways. Post-confluent human PDAC cells were cultured in 24-well plates and incubated in 10% FBS DMEM at 37°C. After washing out the media, cells were incubated in serum-free medium (Conditioned Medium; CM) in the presence or absence of TNF-α at 37°C for 24 hrs. (A) Human PDAC cells were also treated with either 13-cis RA or 1, 25-VD3 in different subgroups. CM was collected and loaded into gelatin-containing zymogram gel stained with Coomassie blue stains (see Materials and Methods). The levels of detection represent zymogram expression of MMP-9 in human PDAC cells. Different upper-case letters (A, B, C) represent statistically significant differences among subgroups in PANC-1 cells (*P*<0.05). Different lower-case letters (a, b, c, d) represent statistically significant differences among subgroups in HPAF-II cells (*P*<0.05). (B) Human PDAC cells were treated with or without TNF-α in the presence of Bay-117082 (a specific NF-κB inhibitor) or SP600125 (a specific JNK inhibitor). Different upper-case letters (A, B) represent statistically significant differences among subgroups in PANC-1 cells (*P*<0.05). Different lower-case letters (a, b, c, d) represent statistically significant differences among subgroups in HPAF-II cells (*P*<0.05). (C) Western blot analysis of cytoplasmic proteins (at 24 hr time point) was performed using monoclonal antibodies against p-JNK (T183/Y185), p-IκB (S32/36) and internal control (actin). Band intensities represent the amounts of p-JNK (T183/ Y185) and p-IκB (S32/36) in cytoplasma (D) Western blot analysis of nuclear proteins (at 24 hr time point) was performed using monoclonal antibodies against p65 NF-κB, c-fos, p-c-jun (S63) and internal control (lamin A). Band intensities represent the amounts of p65 NF-κB, c-fos, p-c-jun (S63) in the nuclei of human PDAC cells.

A recent study suggested that MMP-9 protein was highly expressed in tumor tissues *in vivo* and played a crucial role in the degradation of the surrounding ECM and regulation of cell invasion in PDAC cells [11]. Results from our experiments suggested that treatment of 13-cis RA and 1, 25-VD3 might inhibit TNF-α-mediated cell invasion in human PDAC cells. Thus, we further examined whether 13-cis-RA and 1, 25-VD3 could modulate the expression of MMP-9 protein in human PDAC cells. Our results from zymogram gels demonstrated that TNF-α significantly enhanced the expression of MMP-9 protein up to 5.4 folds in human PDAC PANC-1 and 3 folds in HPAF-II cells, respectively (Fig 3A). In comparison with TNF-α subgroup, cotreatment of 13-cis-RA (1 μM) and 1, 25-VD3 (0.5 μM) effectively reduced the expression of MMP-9 protein about 57.4% in PANC-1 cells and 96% in HPAF-II cells, respectively (Fig 3A). It is plausible that 13-cis-RA and 1, 25-VD3 might block TNF-α−mediated signaling pathways and suppress the cellular level of MMP-9 protein. TNF-α is a cytokine that can modulate the activation of downstream signals including NF-κB and JNK pathways [30, 31]. Therefore, we further examined the molecular mechanisms of action in TNF-α-mediated MMP-9 protein expression in human PDAC cells. As shown in Fig 3B, treatment of either Bay-117082 (a NF-κB specific inhibitor) or SP600125 (a JNK specific inhibitor) could significantly inhibit TNF-α- mediated expression of MMP-9 protein about 67.2% and 84.4% in comparison with TNF-α subgroup in PANC-1 cells, respectively (*P*<0.05). Treatment of either Bay-117082 (a NF-κB specific inhibitor) or SP600125 (a JNK specific inhibitor) could significantly inhibit TNF-α- mediated expression of MMP-9 protein about 85.7% and 75% in comparison with TNF-α subgroup in HPAF-II cells, respectively (*P*<0.05). These results suggested that JNK pathway might play a dominant role in determining the expression of MMP-9 protein in both human PDAC cells.

Thus, we further investigated the inhibitory effects of 13-cis RA and 1, 25-VD3 on TNF-α- mediated activation of downstream JNK and NF-κB signaling pathways. Our results showed that co-treatment of 13-cis-RA and 1, 25-VD3 could significantly inhibit the activation of JNK and NF-κB signaling pathways through decreased phosphorylation levels of JNK and IκBα proteins in the cytoplasm of human PDAC cells (Fig 3C). Moreover, cotreatment of 13-cis RA and 1, 25-VD3 reduced the nuclear levels of Rel A (p65 NF-κB), c-fos and phosphorylated- c-Jun proteins (AP-1 protein) in human PDAC cells (Fig 3D). These results suggested that co-treatment of 13-cis RA and 1, 25-VD3 potentially inhibited TNF-α- mediated expression of MMP-9 protein, in part, through blockade of JNK and NF-κB signaling pathways in human PDAC cells.

### TNF-α suppressed the expression of TIMP3 through modulation of JNK signaling pathway and upregulation of miR221 in human PDAC cells

**Fig 4:**
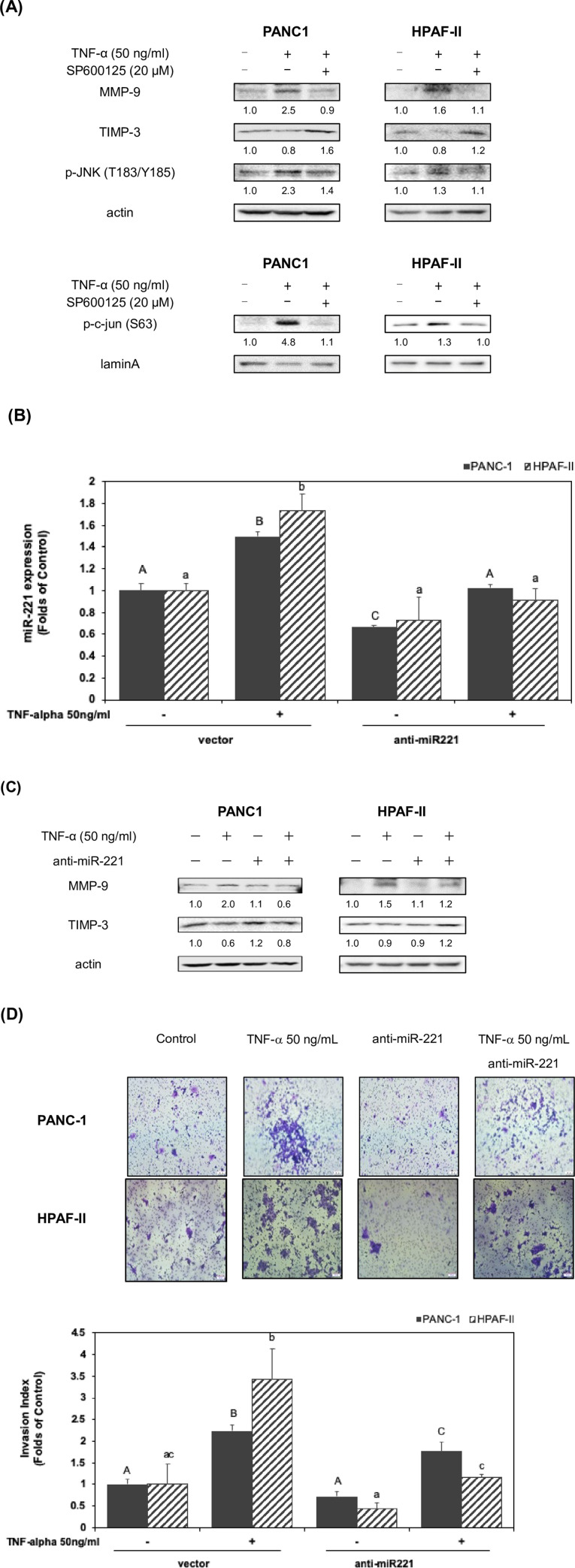
TNF-α suppressed the expression of TIMP3 through modulation of JNK signaling pathway and upregulation of miR221 in human PDAC cells. (A) Human PDAC cells were treated with TNF-α in 10% FBS DMEM in the presence or absence of SP600125 for 24hr. Western blot analysis of cytoplasmic proteins was performed using monoclonal antibodies against TIMP-3, MMP-9 and phosphor-JNK (T183/Y185) and actin, as described under Materials and Methods. Nuclear proteins were performed using antibodies against phosphor-c-jun (S63) and lamin A. Band intensities represent the amounts of TIMP-3, MMP-9, phosphor-JNK and phosphor-c-jun in human PDAC cells. (B) Transfection of human PDAC cells with siRNA against miR 221 (anti-miR 221) was performed as described in Materials and Methods. These human PDAC cells were then treated with or without TNF-α for 24 hr. The effect of TNF-α on the expression of miR 221 was measured by using qPCR assay in the Materials and Methods section. Statistical significance is expressed as the mean ± SD (standard deviation) of two independent experiments. Different upper-case letters (A, B, C) represent statistically significant differences among subgroups in PANC-1 cells (*P*<0.05). Different lower-case letters (a, b) represent statistically significant differences among subgroups in HPAF-II cells (*P*<0.05). (C) Transfection of human PDAC cells with anti-miR 221 siRNA was performed as described in Materials and Methods. These human PDAC cells were then treated with or without TNF-α for 24 hr. Western blot analysis of cytoplasmic proteins (at 24 hr time point) using monoclonal antibodies against MMP-9, TIMP3 and actin. (D) Transfection of human PDAC cells with anti-miR 221 siRNA was performed as described in Materials and Methods. These human PDAC cells were then treated with or without TNF-α for 24 hr. Cell invasion assay was performed as described in the Materials and Methods section. Invasion levels were quantitated and analyzed by using ANOVA analysis. Different upper-case letters (A, B, C) represent statistically significant differences among subgroups in PANC-1 cells (*P*<0.05). Different lower-case letters (a, b, c) represent statistically significant differences among subgroups in HPAF-II cells (*P*<0.05).

A previous study indicated that TIMP3 protein acts as an inhibitor of MMP-9 protein [13]. Our results already indicated that TNF-α induced the expression of MMP-9 mainly through JNK and NF-κB pathways in human PDAC cells (Fig 3B). Our results also demonstrated that JNK pathway seemed to play a pivotal role in determining cellular level of MMP-9 protein in human PDAC cells. Therefore, we examined whether TNF-α could modulate the expression of MMP-9 and TIMP3 proteins through JNK pathway in human PDAC cells. In this study, our results showed that TNF-α induced the expression of MMP-9 protein in human PDAC cells (Fig 4A). Thus, we further investigated whether JNK signaling pathway is involved in the regulation of TIMP3 expression in TNF-α-treated human PDAC cells. Surprisingly, TNF-α inhibited the expression of TIMP3 protein in human PDAC cells (Fig 4A). Treatment of SP600125 significantly induced the expression of TIMP3 in TNF-α-treated human PDAC cells. Our results also confirmed that SP600125 significantly reduced cytoplasmic level of phosphorylated-JNK protein and nuclear level of phosphorylated-c-jun proteins in human PDAC cells (Fig 4A). A previous study demonstrated that TIMP-3 deficient mice promote prostate tumor growth, cell proliferation index, micro-vessel density, invasive capacity and the expression of MMP-9 [12]. These results suggested that treatment of TNF-α lead to a downregulation of TIMP3 expression and an upregulation of MMP-9 protein through the activation of JNK signaling pathway.

Recent studies suggested that the expression of MMP-9 and TIMP3 could be modulated by miR-221 in other human cancer cells [17, 18]. Therefore, we further examined whether TNF-α could modulate the expression of miR-221 in human PDAC cells. As shown in Fig 4B, treatment of TNF-α significantly induced the expression of miR-221 up to 1.5 folds in human PDAC PANC1 cells and 1.7 folds in human PDAC HPAF-II cells. Treatment of siRNA against miR-221 (anti-miR221) would significantly suppress the cellular level of miR-221 in TNF-α-treated cells (*P*<0.05) (Fig 4B). These results suggested that TNF-α might upregulate the expression of miR-221 in human PDAC cells. Therefore, we further investigated whether TNF-α could modulate the expression of MMP-9 and TIMP3 proteins through the regulation of miR-221 in human PDAC cells. As shown in Fig 4C, treatment of siRNA against miR-221 (anti-miR221) lead to a decreased expression of MMP-9 protein and an increment of TIMP3 expression in TNF-α-treated human PDAC cells. These results suggested that TNF-α mediated upregulation of MMP-9 and downregulation of TIMP3 proteins were, in part, through an increased expression of miR-221 in human PDAC cells. To further confirm these findings, we examined whether miR-221 could be involved in TNF-α-mediated cell invasion in human PDAC cells. As shown in Fig 4D, treatment of siRNA against miR-221 (anti-miR221) significantly inhibited TNF-α-mediated cell invasion in human PDAC cells (*P*<0.05). These results suggested that TNF-α-mediated cell invasion, upregulation of MMP-9 and downregulation of TIMP3 proteins were through an increased expression of miR-221 in human PDAC cells.

### 13-cis RA and 1,25-VD3 induced the expression of TIMP3 through a suppression of miR-221 in TNF-α-stimulated PDAC cells

**Fig 5:**
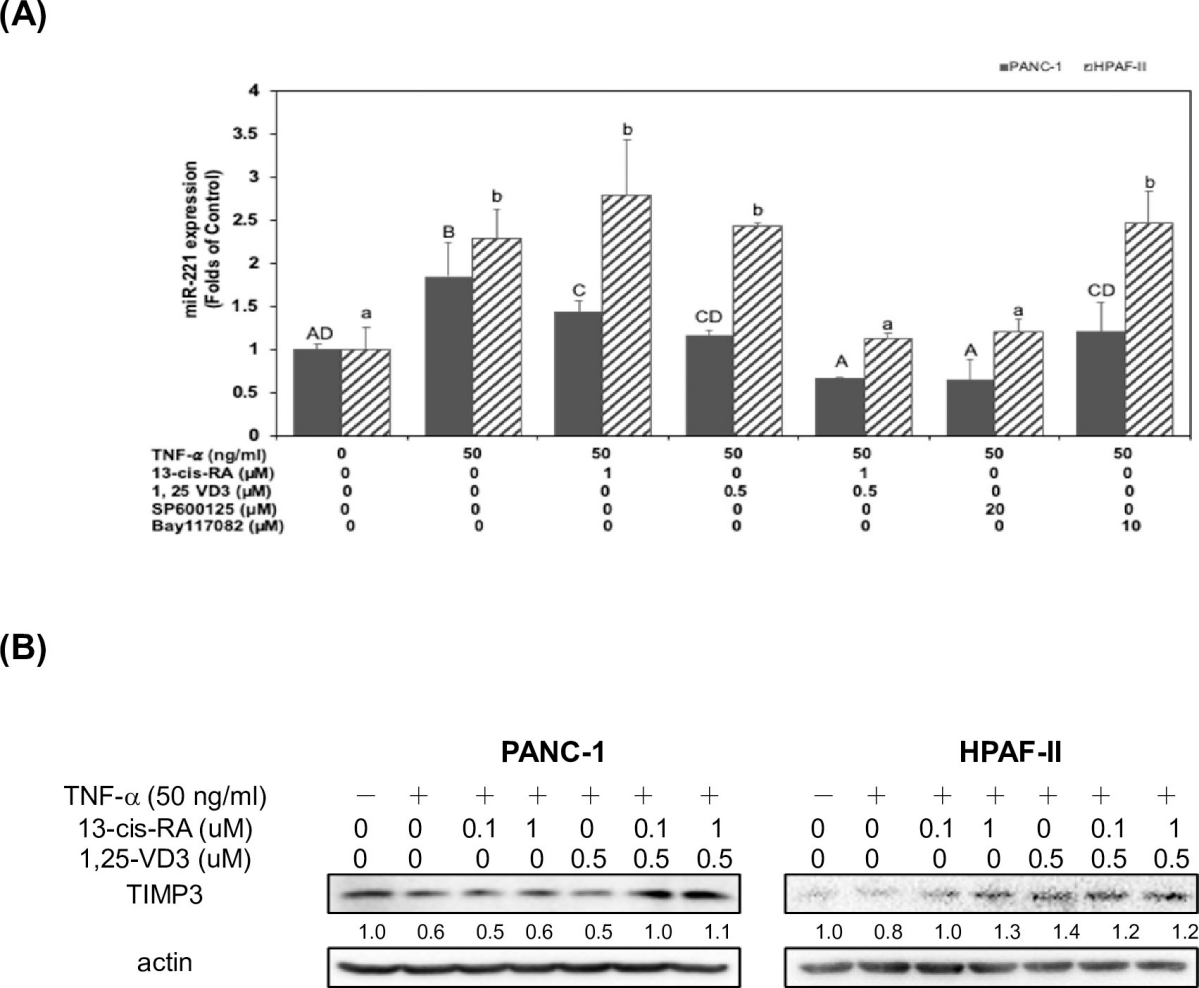
13-cis RA and 1,25-VD3 induced the expression of TIMP3 through a suppression of miR-221 in TNF-α-stimulated PDAC cells. (A) Human PDAC cells were treated with TNF-α in 10% FBS DMEM in the presence or absence of 13-cis RA, 1, 25-VD_3_, SP600125 or Bay117082 for 24hr. The effect of TNF-α on the expression of miR 221 was measured by using qPCR assay in the Materials and Methods section. Statistical significance is expressed as the mean ± SD (standard deviation) of two independent experiments. Different upper-case letters (A, B, C, D) represent statistically significant differences among subgroups in PANC-1 cells (*P*<0.05). Different lower-case letters (a, b) represent statistically significant differences among subgroups in HPAF-II cells (*P*<0.05). (B) Human PDAC cells were treated with TNF-α in 10% FBS DMEM in the presence or absence of 13-cis RA and 1, 25-VD_3_ for 24hr. Western blot analysis of cytoplasmic proteins was performed using monoclonal antibodies against TIMP-3 and actin, as described under Materials and Methods. Band intensities represent the amounts of TIMP-3 in the cytoplasm of human PDAC cells.

Our above results suggested TNF-α could inhibit the expression of TIMP3 and induce the expression of MMP-9 proteins through an induction of JNK pathway and miR-221 expression in human PDAC cells (Fig 4). Our results already demonstrated a pivotal role of JNK pathway in determining cellular level of MMP-9 protein in human PDAC cells (Fig 3B). In this study, we further investigated whether treatment of 13-cis RA and 1, 25-VD3 could reverse TNF-α-mediated suppression of TIMP3 through a reduction of miR-221 expression in human PDAC cells. As shown in Fig 5A, treatment of TNF-α significantly induced the expression of miR-221 in human PDAC cells. Treatment of SP600125 significantly suppressed the expression of miR-221 in TNF-α-treated human PDAC cells. However, treatment of Bay-117082 (a specific NF-κB inhibitor) could not significantly suppress the expression of miR-221 in TNF-α-treated human PDAC cells. These results suggested that TNF-α-mediated expression of miR-221 was through an activation of JNK pathway in human PDAC cells. It is also consistent with the previous findings that miR-221 consists of an AP-1 regulatory binding site. Our results already demonstrated that co-treatment of 13-cis RA and 1, 25-VD3 significantly inhibited TNF-α-mediated activation of JNK pathway and downstream AP-1 proteins (c-fos and p-c-jun proteins) expression human PDAC cells (Fig 3C and 3D). Therefore, we further investigated whether treatment of 13-cis RA and 1, 25-VD3 could modulate the expression of downstream miR-221. As shown in Fig 5A, co-treatment of 13-cis RA and 1, 25-VD3 significantly inhibited the expression of miR-221 in TNF-α−treated human PDAC cells. Our results already showed that co-treatment of 13-cis RA and 1, 25-VD3 significantly inhibited the expression of MMP-9 proteins in human PDAC cells (Fig 3A). These consistent results demonstrated that 13-cis RA and 1, 25-VD3 might suppressed the expression of MMP-9 protein through an inactivation of JNK pathway, decreased expression of AP-1 protein, and downregulation of miR-221 in TNF-α-treated human PDAC cells.

Our results also showed that miR-221 played an important role in determining the expression of TIMP3 protein in TNF-α-treated human PDAC cells (Fig 4C). Therefore, we further examined whether treatment of 13-cis RA and 1, 25-VD3 could modulate the expression of TIMP3 in human PDAC cells. As shown in Fig 5B, co-treatment of 13-cis RA and 1, 25-VD3 significantly enhanced the expression of TIMP3 protein in TNF-α-treated human PDAC cells. Taken together, these results suggested that co-treatment of 13-cis RA and 1, 25-VD3 could upregulate the expression of TIMP3 protein through a blockade of JNK signaling pathway and a decreased expression of miR-221 in TNF-α treated human PDAC cells.

## Discussion

Retinol is a fat-soluble compound which is converted from beta-carotene in plant food and retinyl esters in animal sources. Retinoids (analogues of vitamin A) are converted into retinoic acid (RA) and plays many important roles in the regulation of physiological functions of mammalian cells. For example, retinoid signaling plays a pivotal role in the hindbrain development [32]. Recent studies demonstrated that RA exerts certain roles in the prevention of cancer [33, 34]. A study indicated that 13-cis RA, a RA isomer, improve survival of neuroblastoma patients [35]. Other studies showed a good response of the combined treatment of 13-cis RA and interferon-α in cervical cancer [36]. However, there is no good response from the combined treatment of 13-cis RA and gemcitabine in pancreatic cancer patients [37]. In this study, our results suggested that treatment of 13-cis RA and 1, 25-VD3 could mildly reduce the expression of EMT- regulatory proteins such as Slug, Twist1 and N-cadherin and enhance the expression of E-cadherin protein in PDAC cells (Fig 1). Our results also showed an inducible effect of TNF-α on cell invasion in human PDAC cells. Surprisingly, our results demonstrated that 13-cis RA and 1, 25-VD3 effectively inhibited TNF-α-mediated cell invasion in human PDAC cells (Fig 2A and 2B). Moreover, co-treatment of 13-cis RA and 1, 25-VD3 potentially inhibited TNF-α-mediated expression of MMP-9 protein (Fig 3A). Our results demonstrated that TNF-α enhanced the expression of MMP-9 protein, in part, through the JNK and NF-κB signaling pathways (Fig 3B). 13-cis RA and 1, 25-VD3 inhibited the expression of MMP-9 through a blockade of JNK and NF-κB signaling pathways in human PDAC cells (Fig 3C and 3D).

Several reported that ATRA [26, 38], 9-cis-RA [39], and 13-cis-RA [40] have been tested for their anti-cancer effects in various clinical trials, either alone or combine with other anti-cancer drugs. For example, a study suggested that the cotreatment of 13-cis-RA and histone deacetylase could be a potential method to cure pancreatic cancer [40]. The analogue of vitamin D was also used as an anti-cancer agent for treating pancreatic patients [41]. Previous studies have confirmed that TNF-α promotes the progression and metastasis of many cancer types including hepatic carcinoma [42], hypopharyngeal cancer [43], colorectal cancer [44], and breast cancer [6]. Our results indicated that the co-treatment of 13-cis RA and 1, 25-VD3 significantly inhibited TNF-α -mediated cell invasion (Fig 2A). Surprisingly, our results didn’t show any inhibitory effects of TNF-α on cell survival in human PDAC cells (Fig 2B) and were different from previous reports [45]. One possibility is the differences of culture media (RPMI vs. DMEM media) in the culture of PANC-1 cells. Further evidences needed to be verified in the future study. These results suggested that 13-cis RA and 1, 25-VD3 might act as chemo-preventive agents to prevent cell invasion through targeting specific regulatory cascades in human PDAC cells.

Our results demonstrated that TNF-α- mediated expression of MMP-9 protein might be associated with the JNK or NF-κB signaling pathways. Treatment of SP600125 was generally effective on the suppression of MMP-9 expression in both human PDAC cells (Fig 3B). These results suggested that JNK pathway might play a dominant role in determining the expression of MMP-9 protein in human PDAC cells. Our results showed that co-treatment of 13-cis-RA and 1, 25-VD3 could significantly inhibit the activation of JNK and NF-κB signaling pathways through decreased phosphorylation levels of JNK and IκBα proteins in the cytoplasm of human PDAC cells (Fig 3C). Moreover, cotreatment of 13-cis RA and 1, 25-VD3 reduced the nuclear levels of Rel A (p65 NF-κB), c-fos and phosphorylated- c-Jun proteins (AP-1 protein) in human PDAC cells (Fig 3D). These results suggested that co-treatment of 13-cis RA and 1, 25-VD3 potentially inhibited TNF-α- mediated expression of MMP-9 protein, in part, through blockade of JNK and NF-κB signaling pathways in human PDAC cells.

**Fig 6:**
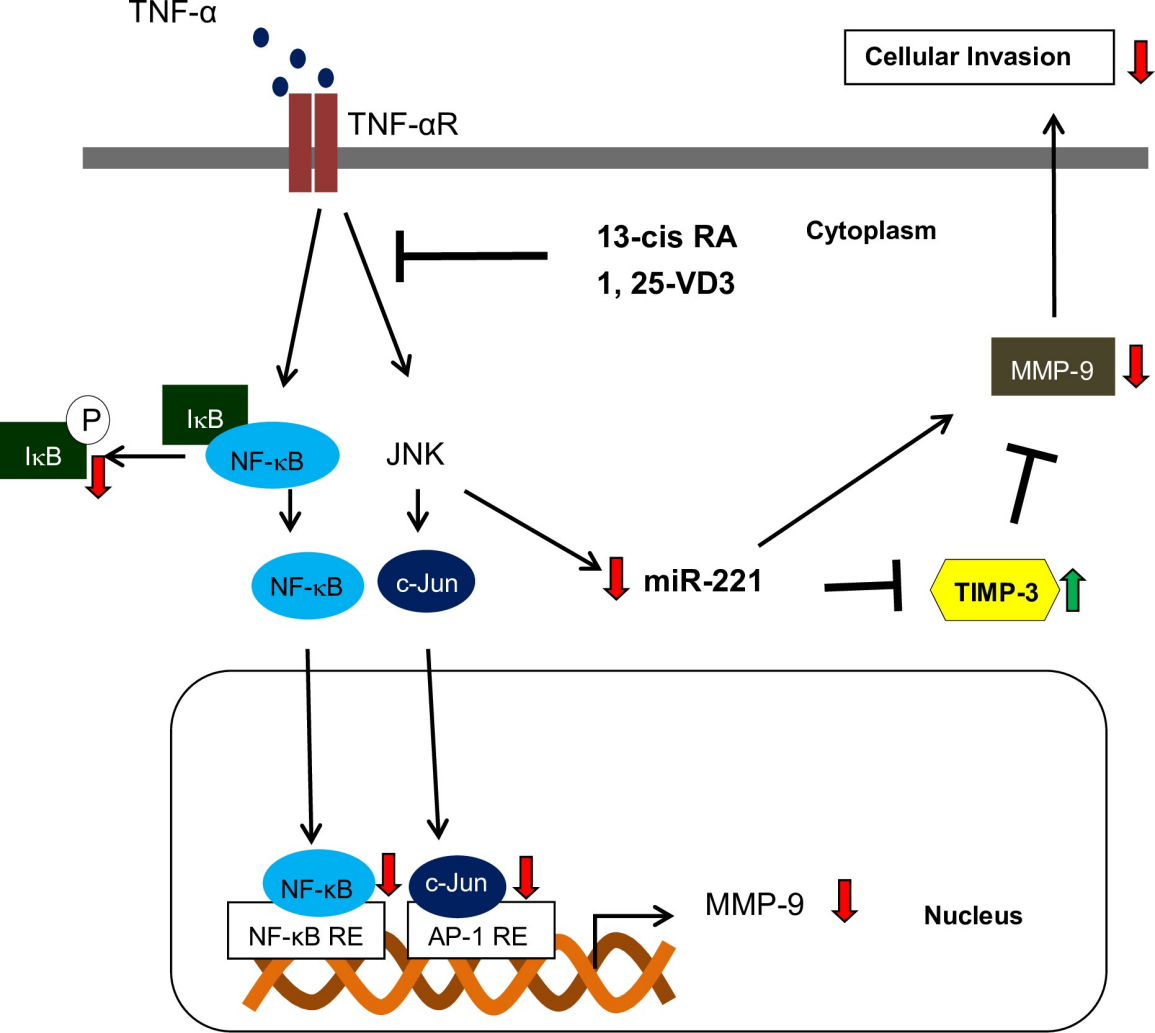
Proposed mechanisms of signaling pathways regulated by 13-cis RA and 1,25-VD3 in TNF-α-treated PDAC cells. Green arrows indicate increase in expression level and red arrows indicate increase in expression level under the cotreatment of 13-cis RA and 1, 25-VD3.: induction;: suppression; RE: response element.

Since TIMP-3 acts as an antagonist of MMP-9, we further examined whether TNF-α could modulated the expression of TIMP-3 protein through the activation of JNK signaling pathway. Our results showed that TNF-α could enhance the expression of MMP-9 but reduce the expression of TIMP-3 protein in human PDAC cells (Fig 4A). Treatment of TNF-α could also increase the expression of miR-221 in human PDAC cells (Fig 4B). Moreover, our results demonstrated that TNF-α-mediated cell invasion, upregulation of MMP-9 and downregulation of TIMP3 proteins were through an increased expression of miR-221 in human PDAC cells (Fig 4C and 4D). These results suggested that miR-221 played an important role in determining the expression of TIMP3 protein in TNF-α-treated human PDAC cells. Moreover, co-treatment of 13-cis RA and 1, 25-VD3 significantly inhibited the expression of miR-221 in TNF-α−treated human PDAC cells (Fig 5A). Therefore, we further examined whether treatment of 13-cis RA and 1, 25-VD3 could modulate the expression of TIMP3 in human PDAC cells. As shown in Fig 5B, co-treatment of 13-cis RA and 1, 25-VD3 significantly enhanced the expression of TIMP3 protein in TNF-α-treated human PDAC cells. Our results already showed that co-treatment of 13-cis RA and 1, 25-VD3 significantly inhibited the expression of MMP-9 proteins in human PDAC cells (Fig 3A). These consistent results demonstrated that 13-cis RA and 1, 25-VD3 might enhance the expression of TIMP3 but reduce the expression of MMP-9 protein through an inactivation of JNK pathway, a decreased expression of AP-1 proteins, and downregulation of miR-221 in TNF-α-treated human PDAC cells. In conclusion, our results demonstrated that 13-cis RA and 1, 25-VD3 could function as chemo-preventive agents to alleviate TNF-α-mediated expression of MMP-9 through blockade of JNK and NF-kB signaling pathways in human PDAC cells. Co-treatment of 13-cis-RA and 1, 25-VD3 further blocked cell invasion and induced the expression of TIMP-3 through an inactivation of JNK pathways and downregulation of miR-221. The probable mechanisms of action were depicted in Fig 6.

## Supporting information

S1 Raw data (PDF)

## Abbreviations

PDAC: pancreatic ductal adenocarcinoma
13-cis RA: 13-cis retinoic acid
1,25-VD3: 1,25-dihydroxyvitamin D3
TNF-α: tumor necrosis factor- α
MMP-9: matrix metalloproteinase-9
TIMP-3: tissue inhibitor of metalloproteinase-3
miR-221: microRNA-221
JNK: c-Jun N-terminal kinase
NF-κB: nuclear factor kappa-B
