# Supplementary material for: Treatment of 13-cis retinoic acid and 1,25-dihydroxyvitamin D3 inhibits TNF-alpha-mediated expression of MMP-9 protein and cell invasion through the suppression of JNK pathway and microRNA 221 in human pancreatic adenocarcinoma cancer cells
Source: PLoS One. 2021 Mar 17;16(3):e0247550. doi: 10.1371/journal.pone.0247550 (PMC7968633; doi:10.1371/journal.pone.0247550)
Supplement: S1 Raw data — (PDF) [file pone.0247550.s001.pdf]

Figure 1.

(A)

	PANC-1	HPAF-II
TNF-α (50 ng/ml)	− + + + + + +	− + + + + + +
13-cis-RA (uM)	0 0 0.1 1 0 0.1 1	0 0 0.1 1 0 0.1 1
1α, 25(OH)$_2$D$_3$ (uM)	0 0 0 0 0.5 0.5 0.5	0 0 0 0 0.5 0.5 0.5
E-cadherin	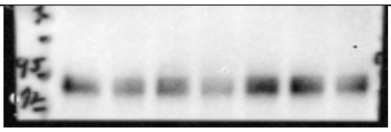	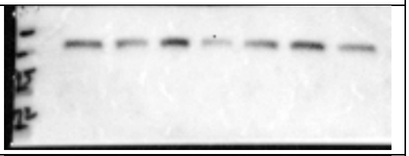
N-cadherin	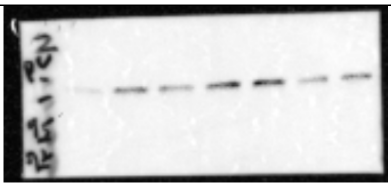	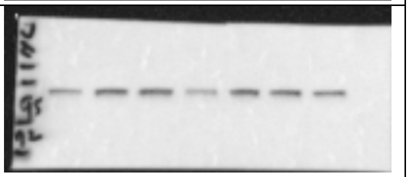
actin	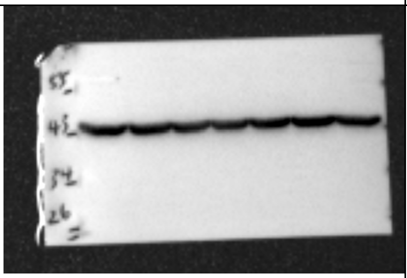	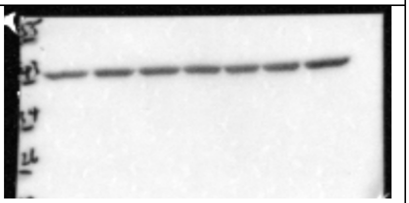

(B)

	PANC-1	HPAF-II
TNF-α (50 ng/ml)	− + + + + + +	− + + + + + +
13-cis-RA (uM)	0 0 0.1 1 0 0.1 1	0 0 0.1 1 0 0.1 1
1α, 25(OH)$_2$D$_3$ (uM)	0 0 0 0 0.5 0.5 0.5	0 0 0 0 0.5 0.5 0.5
Slug	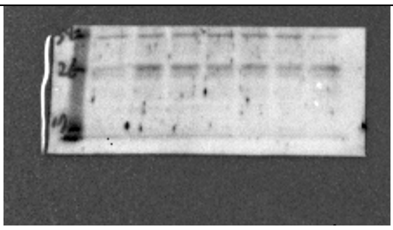	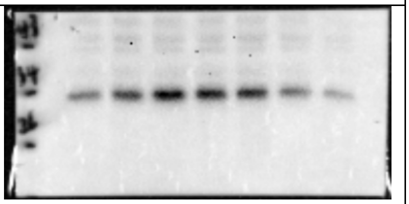
Twist1	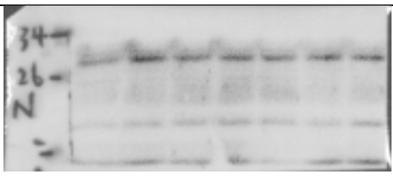	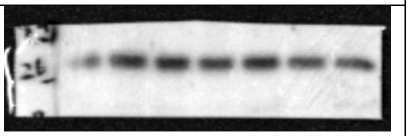
lamin A	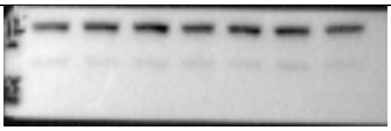	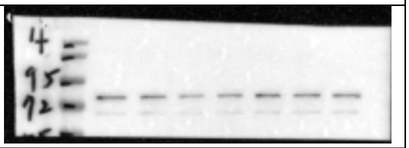

Figure 3.

(A)

	PANC1							HPAF-II						
TNF-α (50 ng/ml)	0	+	+	+	+	+	+	0	+	+	+	+	+	+
13-cis RA (μM)	0	0	0.1	1	0	0.1	1	0	0	0.1	1	0	0.1	1
1,25 VD_3 (μM)	0	0	0	0	0.5	0.5	0.5	0	0	0	0	0.5	0.5	0.5

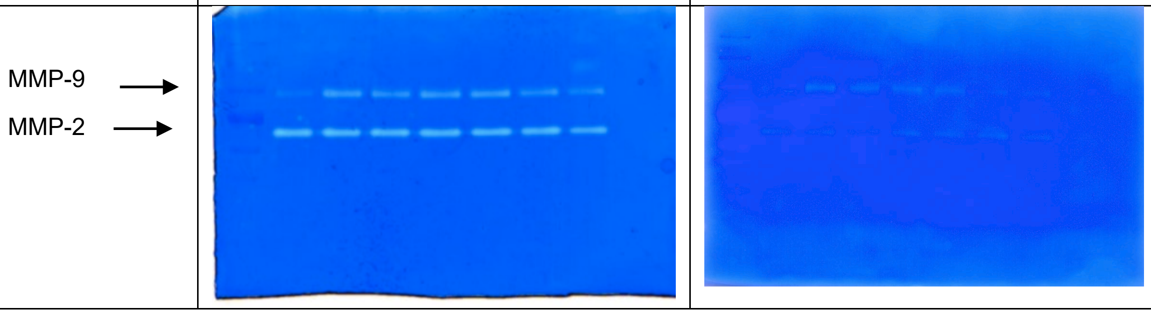

(B)

	PANC-1				HPAF-II			
TNF-α (50 ng/ml)	—	+	+	+	—	+	+	+
Bay-117082 (10 μM)	—	—	+	—	—	—	+	—
SP600125 (20 μM)	—	—	—	+	—	—	—	+

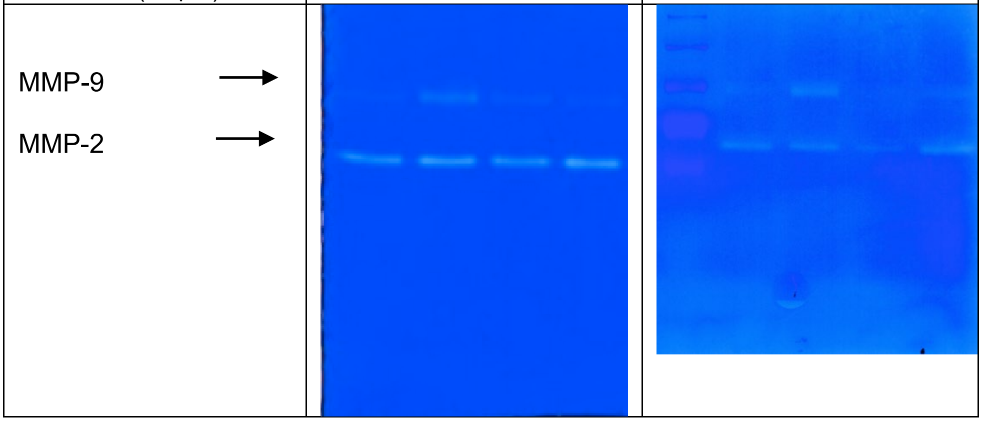

Figure 3.

(C)

	**PANC-1**							**HPAF-II**						
TNF-α (50 ng/ml)	0	+	+	+	+	+	+	0	+	+	+	+	+	+
13-cis-RA (uM)	0	0	0.1	1	0	0.1	1	0	0	0.1	1	0	0.1	1
1,25 VD_3 (uM)	0	0	0	0	0.5	0.5	0.5	0	0	0	0	0.5	0.5	0.5
p-JNK (T183/Y185)	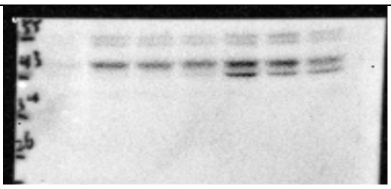							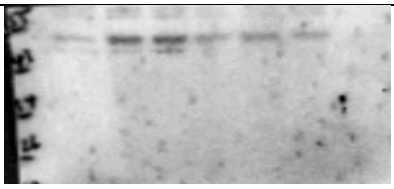						
p-IκBα(S32/36)	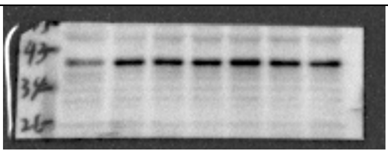							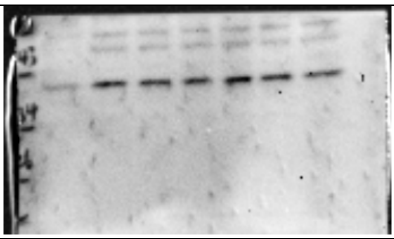						
actin	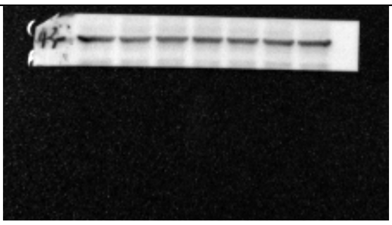							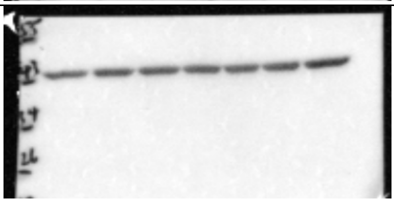						

(D)

	**PANC-1**							**HPAF-II**						
TNF-α (50 ng/ml)	0	+	+	+	+	+	+	0	+	+	+	+	+	+
13-cis-RA (uM)	0	0	0.1	1	0	0.1	1	0	0	0.1	1	0	0.1	1
1α, $25(OH)_2D_3$ (uM)	0	0	0	0	0.5	0.5	0.5	0	0	0	0	0.5	0.5	0.5
p65 NFκB	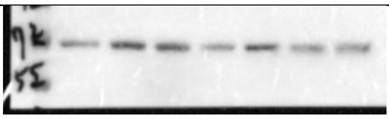							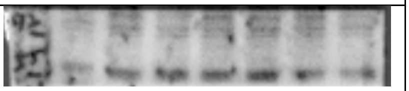						
c-fos	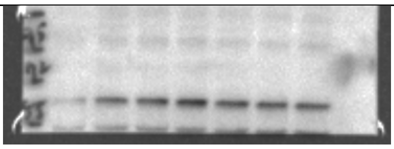							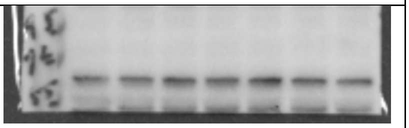						
p-c-jun (S63)	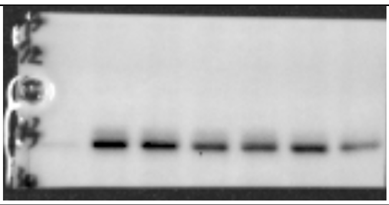							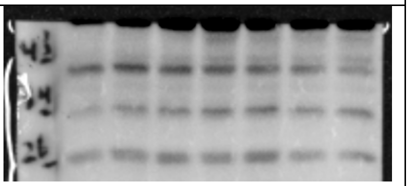						

lamin A	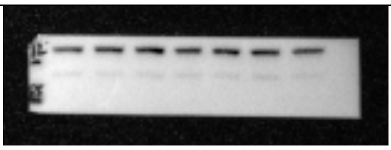	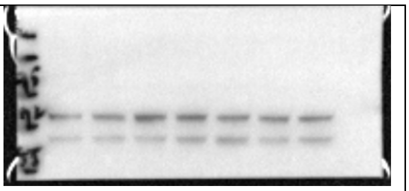

Figure 4.

(A)

	**PANC1**			**HPAF-II**		
TNF-α (50 ng/ml)	−	+	+	−	+	+
SP600125 (20 μM)	−	−	+	−	−	+
MMP-9	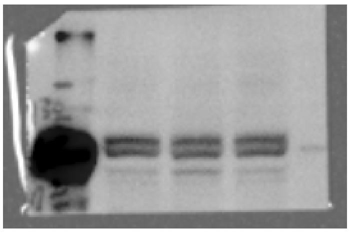			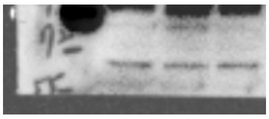		
TIMP-3	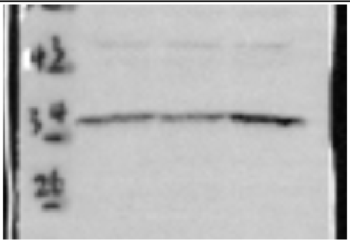			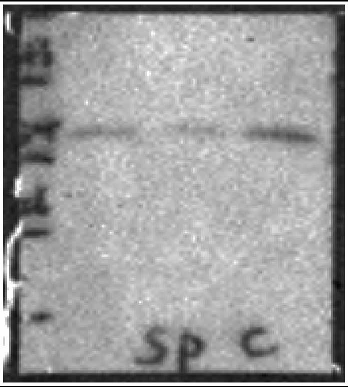		
p-JNK (T183/Y185)	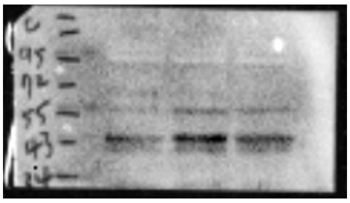			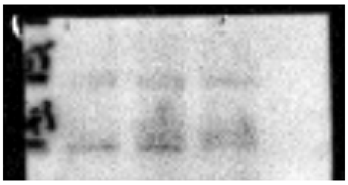		
actin	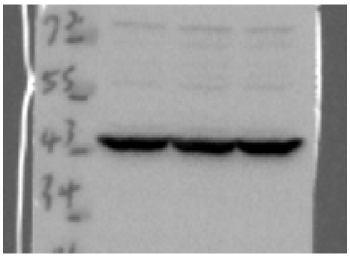			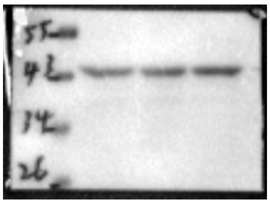		

	**PANC1**			**HPAF-II**		
TNF-α (50 ng/ml)	−	+	+	−	+	+
SP600125 (20 μM)	−	−	+	−	−	+

p-c-jun (S63)	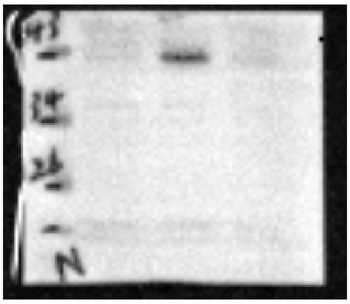	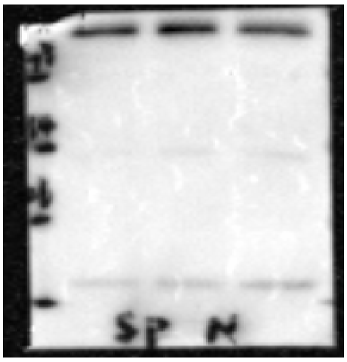
laminA	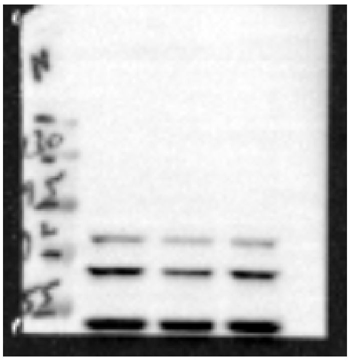	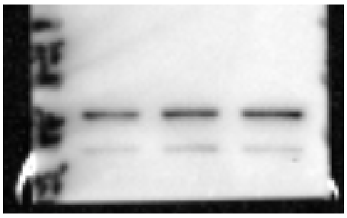

(C)

	**PANC1**	**HPAF-II**
TNF-α (50 ng/ml)	− + − +	− + − +
anti-miR-221	− − + +	− − + +
MMP-9	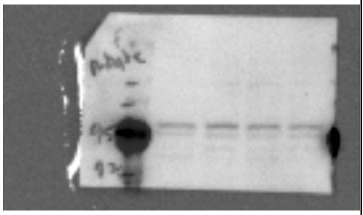	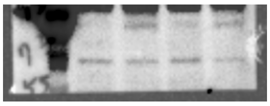
TIMP-3	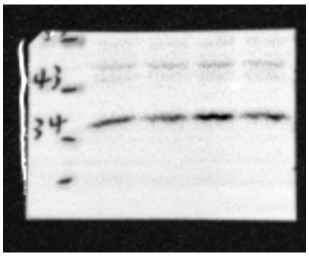	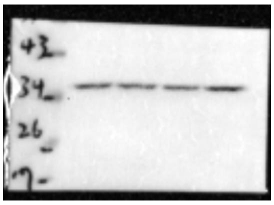
actin	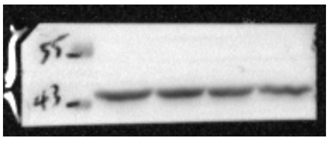	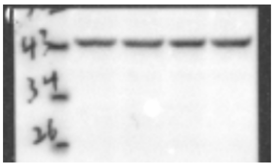

Figure 5.

(B)

	PANC-1							HPAF-II						
TNF-α (50 ng/ml)	−	+	+	+	+	+	+	−	+	+	+	+	+	+
13-cis-RA (uM)	0	0	0.1	1	0	0.1	1	0	0	0.1	1	0	0.1	1
1α, 25$(OH)_2D_3$ (uM)	0	0	0	0	0.5	0.5	0.5	0	0	0	0	0.5	0.5	0.5
TIMP3	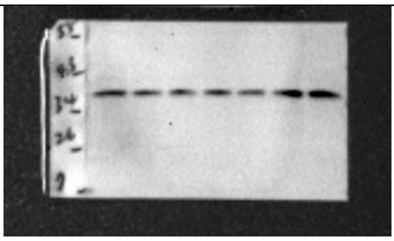							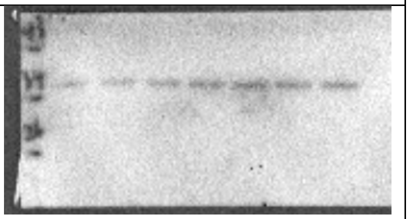						
actin	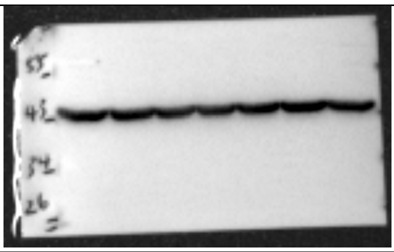							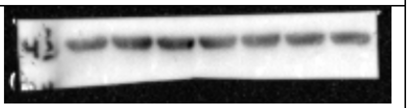						
